# Policies to Reduce Antibiotic Consumption: The Impact in the Basque Country

**DOI:** 10.3390/antibiotics9070423

**Published:** 2020-07-19

**Authors:** Paula Rojas, Fernando Antoñanzas

**Affiliations:** Department of Economics and Business, University of La Rioja, 26004 Logroño, Spain; fernando.antonanzas@unirioja.es

**Keywords:** antibiotics, ARIMA model, co-payment, PRAN, primary care

## Abstract

In 2013, a change in copayment rate was introduced in the Basque Country (one year later than in the other regions in Spain), and improvements were made to drug packaging. In 2014, a National Program Against Bacterial Resistance (Spanish abbreviation: PRAN) was approved. The aim of this study is to analyze the impact of change to the copayment rate, the adjustment of drug packaging, and the approval of PRAN on the consumption of antibiotics. Raw monthly data on the consumption of antibiotics (costs, packages, and daily defined doses per thousand people (DID)) were collected from January 2009 to December 2018 in the Basque Country. Counterfactual and intervention analysis (Autoregressive integrated moving average (ARIMA) model) was performed for the total series, disaggregated by group of antibiotics (2019 WHO Access, Watch, and Reserve (AWaRe) Classification) and active substances with the highest cost per prescription (cefditoren and moxifloxacin), the lowest cost per prescription (doxycycline and cloxacillin), and the most prescribed active ingredients (amoxicillin, azithromycin, and levofloxacin). Introduction of copayment led to a ‘stockpiling effect’ one month before its implementation, equal to 8% in the three consumption series analyzed. Only the adjustment of drug packaging significantly reduced the number of packages dispensed (−12.19%). PRAN approval reduced consumption by 0.779 DID (−4.51%), representing a significant decrease for both ’access’ and ’watch’ group antibiotics. Despite the delay in implementing changes to copayment, there was a ‘stockpiling effect’. With the adjustment of packaging, fewer packs were prescribed but with a higher drug load and price. PRAN approval reduced both the consumption of ’access group antibiotics’ (first-line treatment) and ’watch group antibiotics’ (second-line treatment).

## 1. Introduction

The indiscriminate use of antibiotics accelerates the process of selection and dissemination of bacterial resistance, estimated by the Spanish Society of Infectious Diseases and Clinical Microbiology to lead to 26,000 deaths every year in Spain [1] and to cost €1500 million per year in the European Union [2]. The implementation of regulations to control and monitor the prescription of these drugs is essential in the current context of raising awareness on the use of antibiotics. Changes to pharmaceutical copayment, adjustment of antibiotics packaging (fixing the appropriate number of pills for the most common type of infection), and approval of a National Program Against Bacterial Resistance (PRAN) are three policies recently applied in Spain.

To improve the public health deficit, worsened by the economic crisis of 2008, Royal Decree-Law (RDL) no. 16/2012 was introduced in Spain, containing urgent measures to guarantee the sustainability of the National Health System and the quality and safety of its services [3]. Pharmaceutical copayments became income-based: Pensioner copayment was raised from zero to 10% (with monthly limits of €8.26 and €18.59 for annual incomes below €18,000 and €100,000, respectively), and the working population copayment rose from 40% to 50% on annual income greater than €18,000 and to 60% on annual income over €100,000. Some Autonomous Communities tried to avoid implementing these new pharmaceutical copayments in the belief that the universality of the public health system was not guaranteed. As established by Law 14/1986, in Spain, each Autonomous Community administers a Regional Health Service in order to bring the management of health care closer to the citizen and thus provide guarantees in terms of equity, quality, and participation [4]. The Autonomous Community of the Basque Country managed to delay the application of RDL until July 2013 through Decree no. 114/2012 [5], when the Constitutional Court annulled it in December 2012 [6]. This Autonomous Community of northern Spain has a population of more than two million [7]. Its public health service has a per capita budget of €1731 (year 2019), exceeding the national average by 30.3% and giving the region top position among the Autonomous Communities [8]. Its financing system is different from the other regions since it has its own tax system, so it was possible to delay the entry into force of the RDL until the Supreme Court obliged to implemented in July 2013. There was already a national analysis that estimated that a copayment policy reduced the consumption of medicinal products by 12% (both number of prescriptions and costs) [9], but it is interesting to see if these results are maintained in the Basque Health Service.

The RDL not only made pharmaceutical copayment income-based, but it also introduced regulations to adjust drug packaging to fit the actual duration of the treatment. During the process to approve new medicines, AEMPS, a state agency working as part of the Spanish Ministry of Health since 1999, also evaluates the format in which it is administered. For previously introduced drugs, adjustments were made to the number of units per package [10]. With this objective, in 2012, a legislative measure was approved, the fourth additional provision [11] of RDL no. 16/2012, which changed the format of certain drugs. The aim of this was to ensure that packaging is suitable for the treatment and to reduce economic impact. Drug marketing companies had a period of six months (until January 2013) from the RDL coming into force (July 2012) to withdraw old formats and distribute new ones. However, due to pressure from pharmaceutical companies, this period was extended until May 2013, and even then, drugs could be kept in their old formats until June 2013. This meant that the old formats could no longer be sold from July 2013 [12]. Drugs belonging to therapeutic group J01 (Antibacterials for systemic use) were the first drugs to undergo the review and improvement of their administration formats, based on the recommendations of the health authorities to alter clinical practices to prevent the generation of bacterial resistance.

To reduce the risk of selection and spread of antibiotic resistance, in June 2014, the Inter-territorial Council of the National Health System approved PRAN [13]. This program included six strategic lines in human health, and its objectives focused on monitoring the consumption of antibiotics and raising public awareness. PRAN set a four-year deadline, after which time the effects on consumption should be visible. The effects of this type of program have not been studied in-depth, and few comparisons can be found in literature. Studies monitoring the consumption of antibiotics predominate in different contexts, such as hospitals [14,15] and primary care [16,17,18,19]. All of them conclude with the recommendation not only to introduce health policies to reduce the consumption of these drugs but also to quantify the results. Currently, it is known that introducing these policies significantly reduces the consumption of antibiotics within one year, in both hospital settings [20,21] and primary care [22].

In July 2013, an economic policy, based on changes in copayment rates, and a health policy, based on the adjustment of drug packaging, were applied in the Basque Country. A year later, an additional national program to monitor the consumption of antibiotics was also approved. This study focuses on the Regional Health Service of the Basque Country, following the recommendation by AEMPS to ensure the exploitation and analysis of data on antibiotics consumption at regional level [23]. The objective of this study is to analyze the effect of the change to the copayment rate, the adjustment of drug packaging, and the approval of the PRAN on the consumption of antibiotics, depending on the type of active ingredient prescribed, in the Basque Country.

## 2. Materials and Methods

The database containing all monthly antibiotic prescriptions (therapeutic group J01) in Primary Care from January 2009 to December 2018 in the Basque Country was available. The Basque Government Pharmacy Department provided the data, disaggregated by date of prescription (month and year), active ingredient administered (dose, number of packages, number of prescriptions, and retail price), and patient data (sex, age, and rate of copayment, which is income-based). The doses were given in defined daily doses (DDD). As indicated in the World Health Organization (WHO) methodology, this unit of measurement is subject to continuous variations in order to ensure its representativeness, as it seeks to indicate the maintenance dose in the main indication for a route of administration [24]. DDDs are usually determined for consumption in adults, unless specifically calculated for consumption in children, as is the case for the database used in this study. In order to compare these results with other studies, DDDs were transformed into defined daily doses per thousand people per day (DID), by multiplying by one thousand and dividing then by 365 days times the number of inhabitants. For the population values, data was taken from the Spanish National Institute of Statistics (Spanish acronym: INE) as at July 1 of the corresponding year.

It is important [25] to perform these analyses disaggregating by active ingredient, since differences may depend on the bacterial group. The average cost per prescription in the Basque Country [26] is €12.71: two active ingredients with a high cost per prescription, cefditoren (€43/prescription) and moxifloxacin (€24/prescription), and two active ingredients with a low cost per prescription, doxycycline (€5/prescription) and cloxacillin (€4/prescription), were selected. The most prescribed active ingredient, amoxicillin (representing 23% of all prescribed antibiotics), amoxicillin and beta-lactamase inhibitors (21%), azithromycin (12%), and levofloxacin (5%) were also selected. Except for cefditoren and moxifloxacin, all the drugs were included in the regulations on adjustment to drug packaging. The 2019 AWaRe classification (Access, Watch, and Reserve) issued by the WHO classifies J01 active ingredients in three groups, depending on their probability of generating antibiotic resistance, as follows: ’access group antibiotics‘ for first-line treatment, ’watch group antibiotics‘ for those with a relatively high risk of selection of bacterial resistance, and ’reserve group antibiotics‘ for suspected infections due to multi-drug-resistant organisms only [27]. This classification is used to compare results by groups, with the active ingredients doxycycline, cloxacillin, and amoxicillin belonging to the ’access’ group, and cefditoren, moxifloxacin, azithromycin, and levofloxacin belonging to the ’watch’ group.

As time series were available, analysis was performed according to the Box-Jenkins methodology, frequently [28,29] applied for monthly databases of antibiotic consumption. To estimate the impact of RDL no. 16/2012, counterfactual analysis was performed with the first 53 observations (from January 2009 to May 2013), which allowed for predictions and confidence intervals (80%, commonly applied because it establishes an adequate relationship between precision and width of the interval [30]). If the real value was in the confidence interval of the forecast for the same period, it could be concluded that the policy no longer had a significant effect. Furthermore, the difference between the real value and the predicted value represented the potential savings of the policy under analysis. To understand the impact of the PRAN under approval, intervention analysis was performed that included a dummy variable (V), created to capture the effects of the introduction of RDL no. 16/2012. This variable was at value 1 in June 2013, the month prior to the implementation of copayment, to quantify the stockpiling effect, and the value −1 from July 2013 to the month when the reduction in consumption was no longer significant, according to the results of the previous counterfactual analysis. From January 2009 to June 2015 (one year after approval), 66 observations were made for the intervention analysis.

Box-Jenkins analysis was performed using different packages of R, a software environment for statistical computing and graphics, in order to: represent and break down the variables according to season; obtain the autocorrelation function (ACF) and partial autocorrelation function (PACF), select the best ARIMA model (Autoregressive integrated moving average) according to the Akaike information criterion (AIC), chosen for this study out of the others available (BIC or AICc); and make forecasts for various periods with a confidence level and verify with the Ljung-Box test whether the model adequately captured the information of the observed values and to check that there was no information in the residuals that could be used to make the predictions.

## 3. Results

### 3.1. Copayment and Adjustment of Packaging

To understand the impact of RDL no. 16/2012, counterfactual analysis was performed, which examined the first 53 observations, from January 2009 to May 2013 (Table 1). The predictions obtained for June 2013, the month prior to implementation of the RDL, showed an increase in the administration of antibiotics in the series of costs, number of packages and DID, of 8.31%, 7.21%, and 7.44%, respectively. For the packaging series, all predictions for one year after the policy was applied were significant, resulting in savings of 12.19%. For the costs and DID series, all the observed values fell within the confidence intervals of the predictions, except in July 2013, meaning the policy led to a significant reduction in terms of spending and DID, of 2.20% and 2.07%, respectively (see Appendix A, Table A1, for calculations of savings).

The analysis by active substance showed that, for ’watch’ group drugs, whether they were included in the regulations (azithromycin and levofloxacin) or not (cefditoren and moxifloxacin), there was no significant decrease in the number of packages prescribed. With regard to the active substance of the ’access’ group, on the other hand, the regulation had a significant effect in reducing the number of packages prescribed (see Table A2 for analysis and Table A3 for savings).

### 3.2. PRAN Approval

To estimate the impact of the approval of the PRAN on the consumption of antibiotics, intervention analysis was performed (Table 2) that took into account the first 66 observations, from January 2009 to June 2015 (one year after approval). V1 was used for the cost and DID models, and V2 for the packaging model, taking into account the results of the previous section on the introduction of RDL no. 16/2012. The coefficients of V1 (−0.019 in the cost series and −0.015 in the DID series) and V2 (−0.093 in the packaging series) show that the RDL significantly reduced consumption, corroborating the results obtained in the previous section (see Table A4 for calculations of savings).

The consumption of antibiotics during the year after approval of the PRAN showed a decrease in expenditure by 7.96%, prescribed packages by 8.87%, and the DID by 0.779, mainly with reference to doses of amoxicillin (−0.277 DID), amoxicillin and inhibitors (−0.193 DID) and azithromycin (−0.174 DID), with the latter belonging to the ’surveillance antibiotics‘ group (see Table A5 for analysis and Table A6 for calculations).

**Figure A1:**
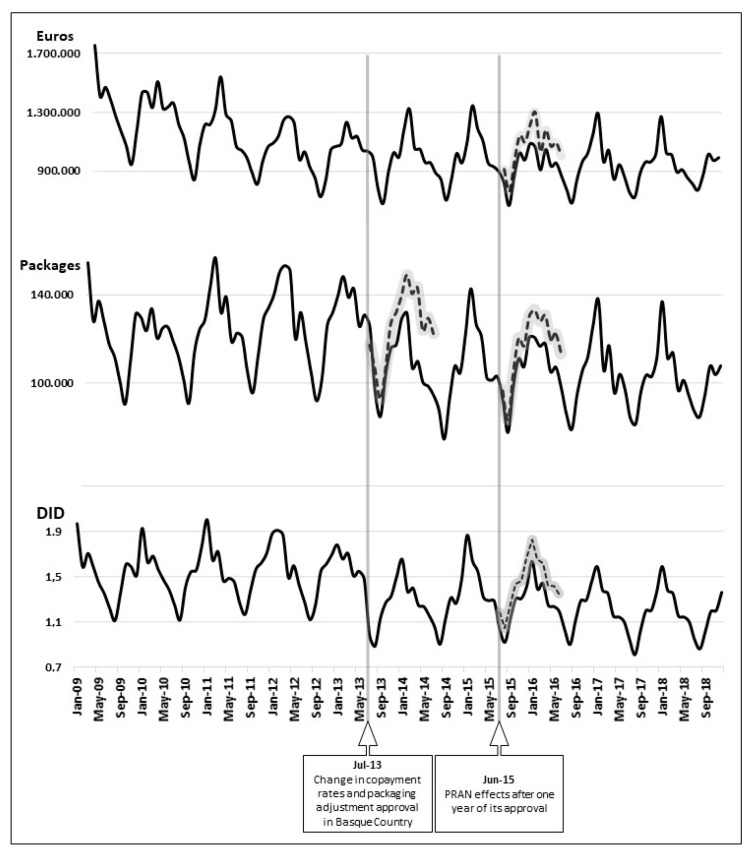
Time series of antibiotic dispensing (€, packages and DID). Black line: monthly prescription values; dashed line: forecasts (80% confidence interval) obtained in counterfactual analysis. Source: Own elaboration based on data provided by the Basque Government Pharmacy Department.

Figure A1 shows the graph of the antibiotic time series expressed in €, packages, and DID. It marks the entry into force of the change in copayment rates, adjustment of packages, and approval of the PRAN. The black line represents monthly prescription values, and the dashed line shows forecasts (80% confidence interval) obtained in counterfactual analysis.

## 4. Discussion

As in other studies [9,31] that found a stockpiling effect prior to implementation of the copayment policy, this analysis also detected an 8% increase in the consumption of antibiotics in June 2013. The Basque Government Health Department issued a statement [26] two months after the copayment implementation, confirming that there had been a stockpiling effect in June 2013, whereby patients purchased 5.03% more drugs compared to June the previous year. As a consequence of applying the RDL, a reduction was only found in the number of packages prescribed, which did not affect DID nor the cost of antibiotics (series that only presented statistically significant reductions in July 2013). According to the Basque Health Department [26], ‘historically, the summer months record lower consumption data than the rest of the year’, but for 2013 these data were significantly higher. Therefore, these results can be attributed to the introduction of the policy to adjust packaging units to fit the treatment, which, despite being an additional provision of the copayment RDL, was only implemented simultaneously with the changes to copayment in the Basque Country. In a study carried out in 20 European countries, copayment schemes were determinant in antibiotic consumption. The purchasing of antibiotics under copayment schemes was 10% lower than in a scheme with full reimbursement system [32]. In addition, in another country-based study, the implementation of a copayment policy had a negative effect of 4% on the consumption of antibiotics for every €1 increase in copayment [33].

Studies on antibiotic dose adjustment are usually placed in a hospital setting. A recent study established that the personalized dosage of antibiotics in hospitalized patients reduces the inappropriate use of these drugs [34]. In this study, the adjustment of packaging was studied in a Primary Care setting. This policy affected ’access group antibiotics’, those that are used as first-line treatment and, therefore, are more frequently prescribed. As indicated before, this decrease in the number of packages prescribed did not translate into a reduction for the other series analyzed; it can therefore be deduced that fewer packages were prescribed, but with a higher drug load and price. This was the only measure that could reduce self-consumption, by preventing the prescribed dose from exceeding the treatment indicated by the healthcare professional, since patients would have a smaller surplus. However, there are no records to quantify the self-consumption of antibiotics. 

In a study undertaken in Belgium, it was found that consumption of antibiotics was reduced by 12.8% DID after an antibiotic awareness campaign was implemented [35]. In other Spanish Regional Services, the consumption of antibiotics was reduced by 5% (both packages and expenditure) after an awareness and rational use campaign was introduced [36]. The first available data on the consumption of antibiotics in Spain coincide with the approval of the PRAN in 2014. According to the PRAN database [37], the consumption of antibiotics in the Basque Country decreased by 0.610 DID, 3.64% from 2015 (16.740 DID) to 2016 (16.130 DID). Our analysis estimated that approval of the PRAN would lead to a reduction of 0.779 DID prescribed, equivalent to a decrease of 4.51% from July 2015 to June 2016. However, this decrease is lower than that estimated for spending and the prescribed quantity expressed in packages (7.96% and 8.87%, respectively). This analysis recorded the greatest reductions in the most frequently prescribed active substances, amoxicillin and azithromycin, classified as ’watch group antibiotics’. It should be noted that DIDs are reviewed annually by the WHO in order to assess their representativeness and that their main objective is to serve as a tool for monitoring drug consumption. When a modification is introduced, the entire time series is corrected so that the values are comparable (in the style of a deflator). In contrast, variations in prices (e.g., generic approval) and packaging (e.g., single-dose packaging) do not make this correction throughout the time series.

One of the strengths of this study is that it performs analysis at the decision-making level. In addition, it uses the Regional Health Authority database of all prescriptions of antibiotics in primary care, which avoids sample-based analysis. One limitation of this study is that the prescription of antibiotics does not correspond to actual consumption (some packages may not be consumed or patient may take leftover drugs). Despite this, antibiotic prescriptions are the closest proxy variable to actual consumption. Another limitation is the use of DDD, which is recommended to monitor consumption in adults, but it has also been applied to pediatric prescriptions as there are no exact values for this group. However, this study included pediatric use as the Basque Health Authority already had internal estimations and provided us with this information.

As a conclusion of this study, it may be pointed out that, despite the delay in applying the copayment, the characteristic stockpiling effect of this type of policy follows the same trend as for the rest of the country. In addition, the adjustment of prescription formats reduced the number of packages, by 12.19%, but did not reduce the trend in either the dose or the expenditure. Finally, the approval of the PRAN effectively reduced the consumption of antibiotics, by 4.51%, not only for those indicated as first-line treatment but also for ’watch group antibiotics’.

## Figures and Tables

**Table 1:** Royal Decree-Law (RDL) no. 16/2012, counterfactual analysis of the series of costs (€), packages, and DID. Real values from January 2009 to May 2013 and predictions from June 2013 to June 2014.

Serie	Total Costs (euros)	Total Packages	Total DID
ARIMA model	(0,0,1) (0,1,1)12	(0,0,1) (0,1,1)12	(0,0,1) (0,1,1)12
MA1	0.519478 *	0.403930 *	0.352600 *
SMA1	0.080095 *	−0.419430 **	−0.446311 **
Q test (*p*-value, delay 18)	10.9600 (0.2785)	8.9238 (0.4443)	8.3222 (0.5020)
AIC	−10.681	−11.744	−11.545
Residual sum of squares	0.0564	0.0796	0.0758
Standard error of the regression	0.0395	0.0447	0.0435
Effect on the series (calculations in Table A1)	Stockpiling effect of 8.31% Savings of 2.20% in expenses, including Jun-13 to Jul-13 (last month with significant effect)	Stockpiling effect of 7.21% Savings of 12.19% in packages, including Jun-13 to Jun-14 (last month with significant effect)	Stockpiling effect of 7.44% Savings of 2.07% in DID, including Jun-13 to Jul-13 (last month with significant effect)

Legend: (*) significant level equal to or less than 0.05; (**) significance level equal to or less than 0.01; ARIMA (integrated autoregressive moving average model); MA1 (first moving average term); SMA1 (first seasonal moving average term); AIC (Akaike information criteria); DID (daily defined doses per thousand people). Source: Own elaboration based on data provided by the Basque Government Pharmacy Department.

**Table 2:** National Program Against Bacterial Resistance (PRAN) approval, intervention analysis of the series of costs, packaging, and DID. Real values from January 2009 to June 2015 and predictions from July 2015 to June 2016 with dummy variables V1 and V2.

Serie	Total Costs (euros)	Total Packages	Total DID
ARIMA model	(0,1,2) (0,1,1)12	(2,0,0) (2,1,0)12	(1,1,2) (0,1,1)12
AR1	-	0.340 ***	−0.423 ***
AR2	-	0.061 *	-
MA1	0.490 ***	-	−0.188 *
MA2	0.381 ***	-	−0.812 *
SAR1	-	−0.446 **	-
SAR2	-	−0.463 ***	-
SMA1	0.754 ***	-	0.772 *
V1	−0.019 *	-	−0.015 *
V2	-	−0.093 **	-
Q test (*p*-value, delay 18)	18.206 (0.252)	18.934 (0.167)	14.701 (0.399)
AIC	−7.138	−7.309	−7.374
Residual sum of squares	0.073	0.076	0.076
Standard error of the regression	0.020	0.017	0.015
Effect on the series (calculations in Table A2)	Savings of 7.96% from Jul. 2015 to Jun. 2016	Savings of 8.87% from Jul. 2015 to Jun. 2016	Savings of 0.779 DID (−4.51% from Jul. 2015 to Jun. 2016)

**Legend:** (*) significant level equal to or less than 0.05; (**) significance level equal to or less than 0.01; (***) level of significance equal to or less than 0.001; ARIMA (integrated autoregressive moving average model); AR1 (first autoregressive term); AR2 (second autoregressive term; MA1 (first moving average term); MA2 (second moving average term); SAR1 (first seasonal autoregressive term); SAR2 (second seasonal autoregressive term); SMA1 (first seasonal moving average term); V1 (dummy variable that takes the value 1 in June 2013 and −1 in July 2013); V2 (dummy variable that takes the value 1 in June 2013 and −1 from July 2013 to June 2014); AIC ( information criterion of Akaike); DID (daily defined doses per thousand people). Source: Own elaboration from data provided by the Basque Government Pharmacy Department.

## Appendix A

**Table A1:** Savings from the introduction of RDL no. 16/2012 (predictions with the ARIMA models in Table 1).

Series	Costs (euros)			Packages			DID		
Date	Real Value	Prediction	Confidence Interval (80%)	Real Value	Prediction	Confidence Interval (80%)	Real Value	Prediction	Confidence Interval (80%)
**Jun. 2013**	994,437 *	918,145 *	843,114–993,176	125,760 *	117,301 *	109,310–125,474	1.286 *	1.197 *	1.111–1.283
**Jul. 2013**	778,627 *	894,975 *	790,709–999,241	95,966 *	106,961 *	98,926–114,997	0.975 *	1.112 *	1.025–1.197
**Aug. 2013**	677,717	716,160	598,665–833,655	84,092 *	92,684 *	84,118–101,350	0.882	0.940	0.849–1.030
**Sep. 2013**	890,812	818,366	700,871–935,862	100,232 *	106,253 *	100,587–114,920	1.131	1.088	0.996–1.179
**Oct. 2013**	1,022,801	1,039,945	922,450–1,157,440	115,925 *	126,973 *	118,307–135,640	1.269	1.295	1.204–1.387
**Nov. 2013**	992,537	1,064,936	947,441–1,182,431	117,578 *	131,980 *	123,313–140,646	1.329	1.337	1.246–1.429
**Dec. 2013**	1,185,331	1,080,010	962,515–1,197,506	129,174 *	139,341 *	130,675–148,007	1.500	1.412	1.320–1.503
**Jan. 2014**	1,320,501	1,231,031	1,113,536–1,348,526	131,722 *	149,125 *	140,461–157,789	1.652	1.562	1.471–1.653
**Feb. 2014**	1,051,123	1,112,522	995,027–1,230,018	106,900 *	140,592 *	131,928–149,255	1.366	1.456	1.365–1.548
**Mar. 2014**	1,047,866	1,126,069	1,008,573–1,243,564	110,981 *	143,465 *	134,802–152,129	1.399	1.484	1.392–1.575
**Apr. 2014**	956,060	1,047,480	929,984–1,164,975	105,361 *	123,841 *	115,177–132,505	1.246	1.283	1.192–1.374
**May 2014**	954,021	1,033,550	916,055–1,151,046	108,572 *	129,514 *	120,851–138,178	1.233	1.318	1.227–1.410
**Jun. 2014**	884,376	1,000,323	882,827–1,117,818	99,485 *	122,447 *	113,783–131,110	1.152	1.240	1.148–1.331
Total (*)	1,773,064	1,813,120		1,431,748	1,630,477		2.261	2.309	
Difference (*)	−40,056			−198,729			−0.048		
Variation (*)	−2.20%			−12.19%			−2.07%		
Stockpiling effect (var. Jun. 2013)	8.31%			7.21%			7.44%		

(*) Only statistically significant predictions are included in the calculation; RDL (Royal Decree-Law); DID (daily defined doses per thousand people). Source: Own elaboration based on data provided by the Basque Government Pharmacy Department.

**Table A2:** Counterfactual analysis of the series of costs and packages by active substance. Real values from January 2009 to May 2013 and predictions from June 2013 to June 2014.

**Active Substance**	**Cefditoren**		**Moxifloxacin**		**Doxycycline**		**Cloxacillin**	
Group	“watch”		“watch”		“access”		“access”	
Chosen by	High cost (€43/recipe)		High cost (€24/recipe)		Low cost (€5/recipe)		Low cost (€4/recipe)	
Included in RDL	No		No		Yes		Yes	
Serie	Costs (euros)	Packages	Costs (euros)	Packages	Costs (euros)	Packages	Costs (euros)	Packages
ARIMA model	(0,0,1) (1,1,0)	(0,0,1) (1,1,0)	(0,0,1) (1,1,0)	(1,0,2) (1,1,0)	(2,0,0) (0,1,1)	(0,1,1) (0,1,1)	(2,0,0) (1,1,0)	(1,0,2) (1,1,0)
AR1	-	-	-	0.48345 *	0.29353 *	-	0.29846 *	0.773629 ***
AR2	-	-	-	-	0.37230 *	-	−0.34330 **	-
MA1	0.41060 **	0.42849 **	0.83520 ***	0.31378 *	-	−0.632895 ***	-	0.369473*
MA2	-	-	-	−0.40898 *	-	-	-	0.150730 *
SAR1	−0.53195 ***	−0.55235 ***	−0.65118 ***	−0.49804 **	-	-	−0.54423 **	−0.503722 *
SMA1	-	-	-	-	−0.56709 *	−0.400906 *	-	-
Q test (*p*-value, delay 18)	7.4520 (0.5962)	7.7591 (0.5586)	7.3600 (0.5997)	7.4616 (0.3824)	10.337 (0.2422)	7.8494 (0.5494)	10.721 (0.218)	8.6277 (0.2805)
AIC	−10.764	−10.130	−11.577	−10.929	−9.03q	−10.878	−8.825	−9.405
Residual sum of squares	0.0604	0.0595	0.0624	0.0605	0.0824	0.0557	0.0793	0.0655
Standard error of the regression	0.0472	0.0443	0.0511	0.0502	0.0712	0.0466	0.069	0.0583
Effect on the series (calculations of savings in Table A3)	not significant		not significant		not significant	Stockpiling effect of 7.63% Packaging reduction of 19.68% including Jun. 2013 to Jun. 2014 (last month with significant effect)	not significant	Stockpiling effect of 7.23% Packaging reduction of 23.62% including Jun. 2013 to Jun. 2014 (last month with significant effect)
**Active Substance**	**Amoxicillin**		**Amoxicillin and Inhibitors**		**Azithromycin**		**Levofloxacin**	
Group	“access”		“access”		“watch”		“watch”	
Chosen by	High prescription (23% of recipes)		High prescription (21% of recipes)		High prescription (12% of recipes)		High prescription (5% of recipes)	
Included in RDL	Yes		Yes		Yes		Yes	
Serie	Costs (euros)	Packages	Costs (euros)	Packages	Costs (euros)	Packages	Costs (euros)	Packages
ARIMA model	(1,1,0) (1,1,0)	(1,0,0) (1,1,0)	(1,0,0) (1,1,0)	(0,0,1) (1,1,0)	(0,1,2) (0,1,1)	(0,1,2) (1,1,0)	(0,1,2) (1,1,0)	(0,0,1) (0,1,1)
AR1	0.75416 **	0.807218 ***	0.822603 ***	-	-	-	-	-
AR2	-	-	-	-	-	-	-	-
MA1	-	-	-	0.524390 ***	−0.12776 *	−0.11887 *	0.44500 ***	0.61486 ***
MA2	-	-	-	-	−0.48812 **	−0.50298 *	0.04571 ***	-
SAR1	−0.57297 **	−0.365231 **	−0.358346 **	−0.55124 **	-	−0.55869 ***	−0.31785 ***	-
SMA1	-	-	-	-	−0.33576 *	-	-	−0.42197 **
Q test (*p*-value, delay 18)	7.6307 (0.5997)	13.476 (0.1422)	12.467 (0.1882)	12.416 (0.1909)	13.514 (0.0953)	14.477 (0.0942)	9.2410 (0.1845)	2.0625 (0.9904)
AIC	−10.895	−11.526	−11.094	−11.118	−9.646	−9.054	−8.016	−17.643
Residual sum of squares	0.0613	0.0650	0.0649	0.0646	0.0708	0.0696	0.0856	0.0545
Standard error of the regression	0.0495	0.0866	0.0848	0.0855	0.0917	0.0864	0.072	0.0387
Effect on the series (calculations in Table A2)	not significant	Stockpiling effect of 9.16% Packaging reduction of 26.79% including Jun. 2013 to Jun. 2014 (last month with significant effect)	not significant	Stockpiling effect of 8.40% Packaging reduction of 26.05% including Jun. 2013 to Jun. 2014 (last month with significant effect)	not significant		not significant	

Legend: (*) significant level equal to or less than 0.05; (**) significance level equal to or less than 0.01; (***) level of significance equal to or less than 0.001; RDL (Royal Decree-Law); ARIMA (integrated autoregressive moving average model); AR1 (first autoregressive term); AR2 (second autoregressive term; MA1 (first moving average term), MA2 (second moving average term); SAR1 (first seasonal autoregressive term); SMA1 (first seasonal moving average term); AIC (Akaike reporting criterion). Source: Own elaboration from data provided by the Basque Government Pharmacy Department.

**Table A3:** Saving of the RDL no. 16/2012 introduction by active substance (predictions with the ARIMA models in Table A2).

**Series** **Active Substance**	**Packages** **Doxycycline**			**Packages** **Cloxacillin**		
**Date**	**Real Value**	**Prediction**	**Confidence Interval (80%)**	**Real Value**	**Prediction**	**Confidence Interval (80%)**
Jun. 2013	1410	1310	1217–1403	2475	2308	2160–2456
Jul. 2013	1009	1302	1210–1,395	2371	2437	2398–2476
Aug. 2013	802	1053	954–1151	2168	2441	2376–2506
Sep. 2013	1211	1319	1215–1423	1985	2469	2383–2555
Oct. 2013	1308	1628	1518–1737	1790	2431	2329–2533
Nov. 2013	1342	1659	1545–1774	1641	2349	2233–2464
Dec. 2013	1234	1543	1424–1662	1536	2281	2154–2407
Jan. 2014	1425	1745	1621–1869	1544	2218	2081–2354
Feb. 2014	1458	1794	1666–1923	1567	2230	2085–2375
Mar. 2014	1490	1902	1769–2035	1565	2242	2089–2395
Apr. 2014	1347	1792	1655–1930	1557	2317	2157–2477
May 2014	1271	1694	1553–1835	1545	2356	2189–2522
Jun. 2014	965	1517	1372–1662	1521	2379	2207–2551
Total	16,272	20,258		23,265	30,458	
Difference	−3986			−7193		
Variation	−19.68%			−23.62%		
Stockpiling effect (var. Jun. 2013)	7.63%			7.23%		
**Series** **Active Substance**	**Packages** **Amoxicillin**			**Packages** **Amoxicillin and Beta-Lactamase Inhibitors**		
**Date**	**Real Value**	**Prediction**	**Confidence Interval (80%)**	**Real Value**	**Prediction**	**Confidence Interval (80%)**
Jun. 2013	34,671	31,763	28,860–34,662	25,897	23,889	22,018–25,760
Jul. 2013	22,177	25,553	22,672–28,433	21,009	24,240	22,400–26,079
Aug. 2013	17,158	21,420	17,719–25,122	19,012	23,343	21,266–25,421
Sep. 2013	24,811	30,277	26,127–34,427	21,377	25,021	22,944–27,098
Oct. 2013	31,244	35,922	31,504–40,340	21,151	28,156	26,079–30,233
Nov. 2013	36,462	41,326	36,742–45,910	18,909	27,648	25,571–29,725
Dec. 2013	31,615	43,703	39,014–48,393	23,170	29,024	26,947–31,101
Jan. 2014	29,702	42,492	37,735–47,248	23,802	32,634	30,557–34,711
Feb. 2014	24,601	43,483	38,684–48,283	18,246	31,123	29,046–33,200
Mar. 2014	25,047	43,360	38,532–48,188	18,852	31,016	28,939–33,093
Apr. 2014	21,518	33,168	28,322–38,014	17,605	26,614	24,536–28,691
May 2014	20,855	37,643	32,785–42,501	17,288	27,619	25,541–29,696
Jun. 2014	20,149	34,337	29,471–39,202	16,864	25,570	23,493–27,647
Total	340,010	464,447		263,182	355,897	
Difference	−124,437			−92,715		
Variation	−26.79%			−26.05%		
Stockpiling effect (var. Jun. 2013)	9.16%			8.40%		

Legend: RDL (Royal Decree-Law). Source: Own elaboration based on data provided by the Basque Government Pharmacy Department.

**Table A4:** Savings from PRAN approval (predictions with the ARIMA models in Table 2).

Series	Costs (euros)			Packages			DID		
Date	Real Value	Prediction	Confidence Interval (80%)	Real Value	Prediction	Confidence Interval (80%)	Real Value	Prediction	Confidence Interval (80%)
Jul. 2015	821,322	866,160	828,657–903,663	92,620	96,947	92,666–101,228	1.137	1.185	1.160–1.211
Aug. 2015	662,357	722,741	672,212–773,270	77,694	83,878	77,715–90,041	1.019	1.049	1.024–1.075
Sep. 2015	864,798	926,602	874,631–978,573	98,640	105,538	98,962–112,115	1.227	1.262	1.237–1.288
Oct. 2015	1,019,837	1,085,420	1,030,836–1,140,004	111,074	120,644	112,646–128,642	1.405	1.438	1.413–1.464
Nov. 2015	972,651	1,042,835	984,719–1,098,951	107,508	117,163	108,265–126,061	1.405	1.458	1.433–1.484
Dec. 2015	1,083,567	1,156,014	1,096,441–1,215,587	120,723	130,513	121,019–140,006	1.513	1.565	1.538–1.592
Jan. 2016	1,060,474	1,234,741	1,172,778–1,296,704	120,736	133,723	123,332–144,115	1.736	1.824	1.797–1.851
Feb. 2016	905,632	981,301	917,010–1,045,592	116,703	127,823	116,757–138,890	1.490	1.64	1.613–1.667
Mar. 2016	1,044,753	1,123,100	1,056,539–1,189,661	117,945	130,498	118,828–142,169	1.540	1.618	1.591–1.645
Apr. 2016	931,082	1,017,023	948,246–1,085,800	105,186	120,087	107,735–132,439	1.345	1.415	1.388–1.442
May 2016	951,560	1,040,102	969,158–1,111,046	107,255	119,942	107,009–132,876	1.335	1.421	1.391–1.451
Jun. 2016	864,528	954,471	881,408–1,027,534	97,951	111,215	97,719–124,711	1.292	1.345	1.315–1.375
Total	11,182,561	12,149,510		1.274.035	1.397.972		16.441	17.220	
Difference	−966,949			−123,937			−0.779		
Variation	−7.96%			−8.87%			−4.51%		

Legend: DID (daily defined doses per thousand people). Source: Own elaboration based on data provided by the Basque Government Pharmacy Department.

**Table A5:** Intervention analysis of the costs and DID series by active principle. Real values from January 2009 to June 2015 and predictions from July 2015 to June 2016 with dummy variable V1.

Active Substance	Amoxicillin		Amoxicillin and Inhibitors		Azithromycin	
Group	“access”		“access”		“watch”	
Chosen by	High prescription (23% of recipes)		High prescription (21% of recipes)		High prescription (12% of recipes)	
**Series**	**Costs (euros)**	**DID**	**Costs (euros)**	**DID**	**Costs (euros)**	**DID**
ARIMA model	(2,0,0) (2,1,0)	(2,1,2) (0,1,1)	(0,1,2) (0,1,1)	(1,0,0) (2,1,1)	(0,1,2) (0,1,1)	(0,1,2) (0,1,1)
AR1	0.438 ***	−0.441 ***	-	0.560 ***	-	-
AR2	0.098 *	−0.292 *	-	-	-	-
MA1	-	−0.151 *	0.270 ***	-	−0.052597 *	−0.040777 *
MA2	-	−0.841 *	0.580 ***	-	−0.547567 ***	−0.638842 ***
SAR1	−0.547 ***	-	-	−0.194 **	-	-
SAR2	−0.405 ***	-	-	−0.374 ***	-	-
SMA1	-	0.845 ***	0.811 ***	0.376 *	−0.521205 ***	−0.577372 ***
V1	−0.047 *	−0.041 *	−0.045 *	−0.040 *	-	-
Q test (*p*-value, delay 18)	15.412 (0.3951)	9.606 (0.7260)	17.420 (0.3214)	15.011 (0.377)	16.517 (0.223)	17.906 (0.1611)
AIC	−7.222	−6.511	−7.182	−7.147	−18.42	−12.45
Residual sum of squares	0.078	0.088	0.091	0.087	0.0615	0.0655
Standard error of the regression	0.017	0.026	0.016	0.018	0.0807	0.0822
Effect on the series (calculations of savings in Table A6)	−9.26%	−0.277 DID (−6.69%)	−10.65%	−0.193 DID (−4.19%)	−14.29%	−0.174 DID (−12.30%)

Legend: (*) significant level equal to or less than 0.05; (**) significance level equal to or less than 0.01; (***) level of significance equal to or less than 0.001; ARIMA (integrated autoregressive moving average model); AR1 (first autoregressive term); AR2 (second autoregressive term; MA1 (first moving average term), MA2 (second moving average term); SAR1 (first seasonal autoregressive term); SMA1 (first seasonal moving average term); V1 (dummy variable that takes the value 1 in June 2013 and −1 in July 2013); AIC (Akaike information criterion); DID (daily defined doses per thousand people). Source: Own elaboration from data provided by the Pharmacy Directorate of the Basque Government.

**Table A6:** Saving of the approval of the PRAN by active substance (predictions with the ARIMA models in Table A5).

**Series** **Active Substance**	**Euros** **Amoxicillin**			**DID** **Amoxicillin**		
**Date**	**Real Value**	**Prediction**	**Confidence Interval (80%)**	**Real Value**	**Prediction**	**Confidence Interval (80%)**
**Jul. 2015**	236,664	257,412	240,284–274,540	0.261	0.271	0.264–0.278
**Aug. 2015**	165,798	186,423	169,294–203,552	0.200	0.247	0.240–0.255
**Sep. 2015**	235,282	255,462	238,332–272,592	0.290	0.300	0.293–0.307
**Oct. 2015**	304,086	326,475	308,695–344,255	0.330	0.341	0.334–0.348
**Nov. 2015**	300,430	319,920	302,140–337,700	0.326	0.343	0.336–0.350
**Dec. 2015**	298,582	318,720	301,588–335,852	0.384	0.393	0.386–0.400
**Jan. 2016**	318,228	338,012	320,878–355,146	0.378	0.487	0.480–0.494
**Feb. 2016**	260,585	342,410	324,672–360,148	0.390	0.399	0.392–0.406
**Mar. 2016**	288,645	311,984	294,246–329,722	0.377	0.399	0.392–0.406
**Apr. 2016**	251,430	281,903	264,165–299,641	0.319	0.328	0.321–0.335
**May 2016**	266,836	291,412	273,674–309,150	0.321	0.334	0.324–0.344
**Jun. 2016**	244,219	264,347	246,609–282,085	0.283	0.294	0.284–0.304
**Total**	**3,170,785**	**3.494.480**		**3.859**	**4.136**	
**Difference**	**−323,695**			**−0.277**		
**Variation**	**−9.26%**			**−6.69%**		
**Series** **Active Substance**	**Euros** **Amoxicillin and Inhibitors**			**DID** **Amoxicillin and Inhibitors**		
**Date**	**Real Value**	**Prediction**	**Confidence Interval (80%)**	**Real Value**	**Prediction**	**Confidence Interval (80%)**
**Jul. 2015**	135,686	143,620	137,669–149,571	0.336	0.349	0.341–0.357
**Aug. 2015**	120,658	134,021	122,677–145,366	0.302	0.316	0.308–0.324
**Sep. 2015**	146,708	160,125	148,329–171,921	0.339	0.360	0.352–0.368
**Oct. 2015**	159,832	174,841	161,689–187,993	0.384	0.396	0.389–0.403
**Nov. 2015**	152,695	170,423	156,503–184,343	0.381	0.394	0.387–0.401
**Dec. 2015**	172,782	191,200	176,314–206,086	0.428	0.448	0.441–0.455
**Jan. 2016**	169,610	187,921	172,230–203,612	0.455	0.468	0.458–0.478
**Feb. 2016**	165,837	184,701	168,181–201,221	0.403	0.420	0.410–0.430
**Mar. 2016**	156,092	177,730	160,440–195,020	0.401	0.420	0.410–0.430
**Apr. 2016**	135,329	158,001	139,953–176,049	0.338	0.354	0.344–0.364
**May 2016**	139,558	161,741	142,967–180,515	0.331	0.351	0.341–0.361
**Jun. 2016**	131,058	154,379	134,898–173,860	0.329	0.347	0.337–0.357
**Total**	**1,785,846**	**1.998.703**		**4.427**	**4,621**	
**Difference**	**−212,858**			**−0.193**		
**Variation**	**−10.65%**			**−4.19%**		
**Series** **Active Substance**	**Euros** **Azithromycin**			**DID** **Azithromycin**		
**Date**	**Real Value**	**Prediction**	**Confidence Interval (80%)**	**Real Value**	**Prediction**	**Confidence Interval (80%)**
**Jul. 2015**	76,836	86,747	77,667–95,826	0.081	0.089	0.082–0.097
**Aug. 2015**	63,910	76,970	64,463–89,477	0.066	0.078	0.067.20 0.090
**Sep. 2015**	81,021	95,662	82,639–108,685	0.087	0.101	0.089–0.112
**Oct. 2015**	95,210	110,230	96,711–123,750	0.101	0.115	0.103–0.127
**Nov. 2015**	95,016	109,752	95,754–123,751	0.090	0.104	0.092–0.117
**Dec. 2015**	119,522	135,372	120,911–149,834	0.123	0.139	0.126–0.152
**Jan. 2016**	128,086	154,055	139,144–168,965	0.144	0.159	0.145–0.172
**Feb. 2016**	118,522	135,161	119,815–150,507	0.123	0.139	0.126–0.153
**Mar. 2016**	112,412	130,740	114,971–146,510	0.119	0.135	0.121–0.150
**Apr. 2016**	96,023	112,525	96,344–128,707	0.102	0.117	0.103–0.132
**May 2016**	94,177	112,275	95,691–128,859	0.101	0.117	0.102–0.132
**Jun. 2016**	94,204	111,289	94,313–128,265	0.098	0.114	0.099–0.129
**Total**	**1,174,938**	**1,370,780**		**1.24055**	**1.414558**	
**Difference**	**−195,841**			**−0.17401**		
**Variation**	**−14.29%**			**−12.30%**		

Legend: DID (daily defined doses per thousand people). Source: Own elaboration based on data provided by the Basque Government Pharmacy Department.
